# Identifying microRNAs Possibly Implicated in Myalgic Encephalomyelitis/Chronic Fatigue Syndrome and Fibromyalgia: A Review

**DOI:** 10.3390/ijms25179551

**Published:** 2024-09-03

**Authors:** Maria Tsamou, Fabiënne A. C. Kremers, Keano A. Samaritakis, Erwin L. Roggen

**Affiliations:** ToxGenSolutions (TGS), 6229 EV Maastricht, The Netherlands; fabienne.kremers@toxgen.solutions (F.A.C.K.); keano.samaritakis@toxgen.solutions (K.A.S.); erwin.roggen@toxgen.solutions (E.L.R.)

**Keywords:** miRNA, chronic fatigue syndrome, fibromyalgia, chronic pain, chronic disease

## Abstract

Myalgic encephalomyelitis/chronic fatigue syndrome (ME/CFS) and fibromyalgia (FM) are chronic syndromes of unknown etiology, accompanied by numerous symptoms affecting neurological and physical conditions. Despite frequent revisions of the diagnostic criteria, clinical practice guidelines are often outdated, leading to underdiagnosis and ineffective treatment. Our aim was to identify microRNA (miRNA) biomarkers implicated in pathological mechanisms underlying these diseases. A comprehensive literature review using publicly accessible databases was conducted. Interesting miRNAs were extracted from relevant publications on ME/CFS and/or FM, and were then linked to pathophysiological processes possibly manifesting these chronic diseases. Dysregulated miRNAs in ME/CFS and FM may serve as promising biomarkers for these diseases. Key identified miRNAs, such as miR-29c, miR-99b, miR-128, miR-374b, and miR-766, were frequently mentioned for their roles in immune response, mitochondrial dysfunction, oxidative stress, and central sensitization, while miR-23a, miR-103, miR-152, and miR-320 were implicated in multiple crucial pathological processes for FM and/or ME/CFS. In summary, both ME/CFS and FM seem to share many dysregulated biological or molecular processes, which may contribute to their commonly shared symptoms. This miRNA-based approach offers new angles for discovering molecular markers urgently needed for early diagnosis or therapeutics to tackle the pathology of these medically unexplained chronic diseases.

## 1. Introduction

Among chronic diseases, fibromyalgia (FM) and myalgic encephalomyelitis/chronic fatigue syndrome (ME/CFS) have been estimated to affect around 1.0–2.7% of the global population [1,2]. The prevalence of both FM and ME/CFS is higher in women (4.0% and 1.7%, respectively) than in men (2.4% and 0.9%, respectively) [1,3,4], especially in middle-aged individuals between 30 and 50 years old [5]. Due to the lack of medical education programs and the outdated guidance for practicing clinicians, these diseases are not accurately diagnosed [6,7,8], adversely affecting not only the quality of life of the patients, but also the associated costs incurred by healthcare systems.

There is a substantial clinical overlap between ME/CFS and FM. Both ME/CFS and FM are chronic syndromes of unknown etiology, characterized by neurological and physical conditions, which commonly share the symptoms of persistent debilitating fatigue and chronic pain that are medically unexplained for more than 3 months in the case of FM and more than 6 months for ME/CFS [9]. Multisystem symptoms have been described for these chronic diseases, including extreme fatigue, myalgia (muscle and joint pain), post-exertional malaise (PEM), brain fog, cognitive dysfunction, disturbed sleep patterns, anxiety, depression, mood disorders, and gastrointestinal disturbances [10,11]. Based on the Institute of Medicine (IOM) diagnostic criteria for ME/CFS (2015), three symptoms (extreme fatigue, PEM, and disturbed sleep) and at least one of two additional manifestations (cognitive impairment and orthostatic intolerance) are prerequisites for diagnosis [12]. As for FM diagnosis, according to the American College of Rheumatology (ACR) diagnostic criteria (2016), bilateral pain on the left and right sides of the body both above and below the waist and chronic generalized pain (on the cervical spine, anterior chest, thoracic spine, or low back) in at least 11 of the 18 tender points should be detected [13,14]. Several risk factors have been implicated in the onset of these chronic diseases [15]. These include genetic and family predisposition [16], environmental factors related to toxins [17], toxic metals [18], (agricultural) chemicals [19,20,21], and viral and bacterial infections [17,22,23,24,25]. Additionally, autoimmunity [26,27], stress conditions [28], and nutritional deficiencies [29,30] may also play significant roles.

A large body of evidence on the potential pathological mechanisms underlying either ME/CFS or FM supports the notion that the multiple system dysregulation of the central nervous system (CNS), immune system, metabolism, and ion transport may play a role in these chronic-fatigue-related diseases [10]. Importantly, these diseases are overrepresented by women, exhibiting an apparent gender/sex-specific pattern in disease susceptibility. Although many studies have invested substantial efforts into better understanding the pathophysiological basis of these chronic diseases and to improve their treatment, their etiology and even their diagnosis remain undetermined. There is an urgent need for biomarkers that can efficiently detect these diseases at an early stage and contribute to effective personalized treatments to alleviate pain and improve patients’ clinical conditions.

MiRNAs are short (18–21 nucleotides) non-coding RNA molecules known as master regulators of gene expression that control more than 60% of the human genome, binding to a specific sequence at the 3-untranslated region (3-UTR) of their target genes and inducing translational repression [31]. MiRNAs regulate a wide range of cellular and biological processes [32]. Several miRNAs have been suggested as promising biomarkers for many pathological conditions. Evidence has reported aberrant miRNA expression in chronic diseases [33,34]. Identifying candidate miRNA biomarkers implicated in the underlying mechanisms of these chronic fatigue syndromes is of great importance. 

This review aims to strengthen the current understanding of the complex etiology and challenging diagnosis of FM and ME/CFS, with a particular focus on emerging miRNA biomarkers. We linked the identified miRNAs with dysregulated biological and cellular processes that have been implicated in the pathophysiology of FM and ME/CFS. This may help to develop more targeted and effective therapeutic strategies. Ultimately, by using this mechanistic-based approach, we aim to bridge the gaps in current research and suggest innovative directions for future studies. This, in turn, could improve both the diagnosis and management of FM and ME/CFS and improve the quality of life of affected individuals.

## 2. Methods

### Literature Review for miRNAs in ME/CFS and FM

**Figure 1:**
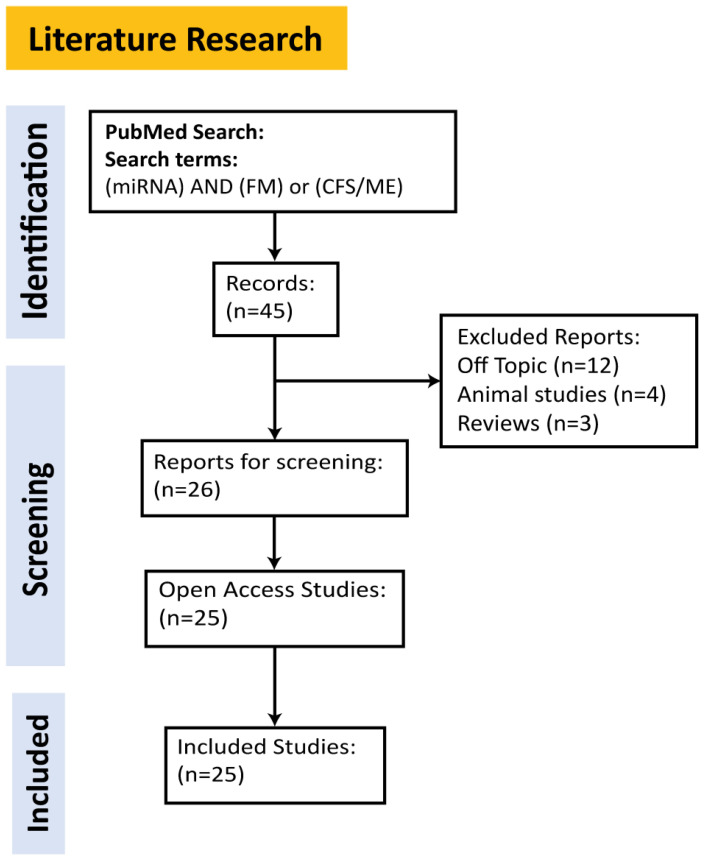
Flow diagram of the literature screening process for the studies included in our review.

For the selection of miRNAs relevant to the chronic conditions of interest, we performed a literature search using public bibliographic databases (PubMed). Our research strategy included the keywords “miRNA”, “chronic fatigue syndrome (CFS)”, “myalgic encephalomyelitis (ME)”, and “fibromyalgia (FM)”. We applied specific search filters to refine the results, limiting the selection to studies that were open access, published after 2010, available in English, and focused on human studies. Additionally, “off-topic” and retracted articles were excluded from consideration. The final set of selected articles (*n* = 25) had publication dates prior to 10 January 2024. A visual representation of our literature review process is provided in Figure 1. 

## 3. Discussion

### 3.1. Dysregulated Biological and Cellular Processes Shared by ME/CFS and FM

Numerous studies have shown that abnormal changes in the immune system [35], chronic inflammatory pathways and oxidative stress pathways [36,37], mitochondrial dysfunction [38], reduced vascular function [39,40], tryptophan metabolism [41], transient receptor potential (TRP) ion channel disorder [42], and central sensitization [43] may be involved in both ME/CFS and FM. Additionally, alterations in the autonomic and neuroendocrine system involving the Hypothalamic–Pituitary–Adrenal (HPA) axis, the activation of cell signaling networks, and disturbances in metabolic pathways have also been implicated [44,45]. These factors contribute to the common symptoms of these conditions, such as chronic (widespread) pain, chronic fatigue, memory deficits, and depression [46,47].

In more detail, the *dysregulation of the immune system* is considered to contribute to the etiology of chronic fatigue diseases, inducing changes in cytokine profiles, immunoglobin levels, and the function of T and B cells and natural killer (NK) cells [30,48,49,50,51]. In particular, in ME/CFS, several immunological abnormalities including the impaired cytotoxic function of NK cells and CD8+ T cells, the presence of autoantibodies mainly targeting the central nervous system (CNS), and increased levels of proinflammatory cytokines have been described [52]. T cells have also been implicated in pain development and central sensitization, suggesting that numerous T cell subpopulations, including CD3+ T cells, CD4+ T helper cells, and CD8+ cytotoxic T cells, influence the pathophysiology of FM [48]. Increased levels of systemic and neuroinflammatory markers have been seen in both FM and ME/CFS [35,53]. Theoharides et al. [54] suggested that the role of mast cells as an “immune gate to the brain”, which can be stimulated by neuropeptides such as substance P, known to be increased in the cerebrospinal fluid (CSF) of FM patients [55], or sex hormones such as estrogens, possibly accounts for women’s overrepresentation in FM and CFS/ME [56,57,58]. 

In both ME/CFS and FM, *mitochondrial dysfunction* and reduced ATP levels in muscle and neural cells have been attributed to the widespread musculoskeletal pain seen in FM and sometimes in ME/CFS [38]. An imbalance between oxidants and antioxidants has been linked to these chronic diseases [36,37], supporting the role of *oxidative stress* in the development and progression of the diseases. Morris et al. [59] suggested that increased Nuclear Factor kappa B (NF-kB), causing the release of pro-inflammatory cytokines, and decreased tumor suppressor protein p53, causing aerobic mitochondrial dysfunction, are the key mechanisms in ME/CFS associated with the increased production of reactive oxygen species (ROS), mitochondrial exhaustion, and the additional need for ATP production.

A deficiency in the CoQ10 enzyme, which has been reported in both ME/CFS and FM [60,61], can lead to mitochondrial dysfunction because of the decreased activity of mitochondrial respiration and mitochondrial membrane potential, resulting in ROS production and, subsequently, in the selective degradation of mitochondria mediated by *autophagy*, known as mitophagy [62]. Autophagy is an important cellular process that involves the degradation of damaged cytosolic components by lysosomes and ensures balance in energy sources [48]. Changes in autophagic genes or proteins have been reported in FM [61] and ME/CFS [63].

Growing evidence supports the implication of *reduced vascular function* in both FM [39] and ME/CFS [40]. Endothelial dysfunction, which refers to the abnormal function of endothelial cells likely leading to decreased vasodilation along with reduced nitric oxide (NO) (endothelium-relaxing mediator) availability, has been associated with the severity of symptoms of these chronic diseases [64,65]. The decreased post-occlusive hyperemic response of branchial artery and forearm skin microcirculation were observed in ME/CFS patients compared to healthy controls [66]. The reduced brachial artery flow-mediated dilatation in FM patients in response to increased blood flow or NO has been attributed to the unavailability of NO to smooth muscle cells, to muscle cell dysfunction, or to the inability of the arteries to relax [65].

Also, *TRP ion channel disorder* has been linked to chronic fatigue. The TRP ion channels, activated by G-protein-coupled receptors (GPCRs), are non-selective cation channels that possess high permeability for calcium (Ca^2+^) implicated in store-operated calcium entry (SOCE) in the white matter of the CNS, and their dysfunction can lead to reduced intracellular Ca^2+^ mobilization [67,68]. TRP ion channels are susceptible to stressors such as viruses and have a key role in the regulation of Ca^2+^ signaling, which is important for cell functions, intracellular signaling pathways, and cellular homeostasis [42].

Chronic widespread pain has been described as a consequence of *central sensitization*, which is characterized by the increased and abnormal responsiveness of nociceptive neurons to a variety of stimuli, leading to disturbed pain processing in the CNS [43]. The activation of ionotropic N-methyl-D-Aspartate (NMDA), a-amino-3-hydroxy-5-methyl-4-isoxazole propionate (AMPA) receptors, metabotropic glutamate receptor subtypes (mGluR), brain-derived neurotrophic factor (BDNF), substance P and calcitonin gene-related peptide (CGRP), NO, and bradykinin have been implicated in initiating and maintaining central sensitization activity and pain hypersensitivity [69]. Also, the activation of Toll-like receptors (TLRs), such as increased TLR-4 signaling in glia and sensory neurons, has been implicated in the sustained proinflammatory condition within spinal cord and neuronal central sensitization, affecting the nociceptive pathways and thereby resulting in persistent pain [70,71]. 

Central *HPA dysfunction* has been implicated in stress-related disorders including ME/CFS and FM [44,45]. In ME/CFS and FM, several potential mechanisms have been described as underlying hypercortisolism or hypocortisolism, characterized by high or low levels of circulating cortisol (corticosteroids), respectively, which involve the release of neurohormones, corticotropin-releasing hormone (CRH), and arginine vasopressin (AVP) from the hypothalamus, stimulating their receptors and leading to the release of adrenocorticotropic hormone (ACTH) from the pituitary into systemic circulation, affecting the release of glucocorticoids from the adrenal cortex [45,72,73]. Cortisol acts via a negative feedback mechanism, causing a decrease in the secretion of CRH, AVP, and ACTH [74]. During chronic stress, the sustained activation of the HPA axis has been described [45]. Also, under chronic inflammatory conditions, a shift from CRH to AVP dominance in HPA activity has been described [75].

*Tryptophan metabolism* mediates the interactions among the blood, brain, and immune system [41]. Tryptophan breaks down via the methoxyindole pathway (1%), leading to the production of serotonin (5-hydroxytryptamine, 5-HT), or via the kynurenine (KYN) pathway (>95%), leading to the de novo synthesis of nicotinamide adenine dinucleotide (NAD+). In the KYN pathway, tryptophan 2,3-dioxygenase (TDO) is stimulated by cortisol, while indoleamine 2,3-dioxygenases (IDOs) are upregulated by lipopolysaccharides and proinflammatory cytokines and downregulated by antioxidants and anti-inflammatory cytokines [76]. Among the KYN metabolites, quinolinic acid (QA) is the most neurotoxic; it decreases NAD levels and acts as an agonist of NMDAR, causing neuronal and astrocytic damage by increasing the intracellular Ca^2+^. Kynurenic acid (KYNA) is neuroprotective; it is produced by astrocytes and acts as an antagonist of NMDAR [41]. Tryptophan dysmetabolism via the KYN pathway and its metabolites, mainly IDOs, 5-HT, KYNA, and QA, may contribute to fatigue, gastrointestinal disorders, depression, neuroinflammation, cognitive dysfunction, brain fog, sleep disorders, and immune system abnormalities [41], all of which have been described as shared symptoms by ME/CFS and FM.

Lastly, metabolic syndrome and obesity have been associated with symptom severity in chronic fatigue disorders [77]. Women with FM were found to be 5.5 times more likely than healthy women to acquire metabolic syndrome and have a larger waist circumference [78]. In FM, a high prevalence (17.3%) of deficiency in growth hormone (GH)/insulin-like growth factor 1 (IGF1) signaling in obesity has also been described [79,80,81]. In women with FM, higher levels of total and low-density cholesterol have been reported, as well as higher levels of glycosylated hemoglobin and triglycerides in serum and higher levels of norepinephrine (NE)/epinephrine and NE/cortisol ratios in urine, leading to higher systolic and diastolic blood pressure [78]. Finally, an altered fatty acid and lipid metabolism was revealed by metabolic profiling in the plasma of ME/CFS patients, indicating disturbances in taurine and glycerophospholipid metabolism among other fatty-acid-metabolism-related pathways, as well as in purines (ADP and ATP), pyrimidines, amino acids, glucose, and oxaloacetate metabolic pathways [82].

### 3.2. miRNAs Potentially Implicated in FM and/or ME/CFS

**Figure 2:**
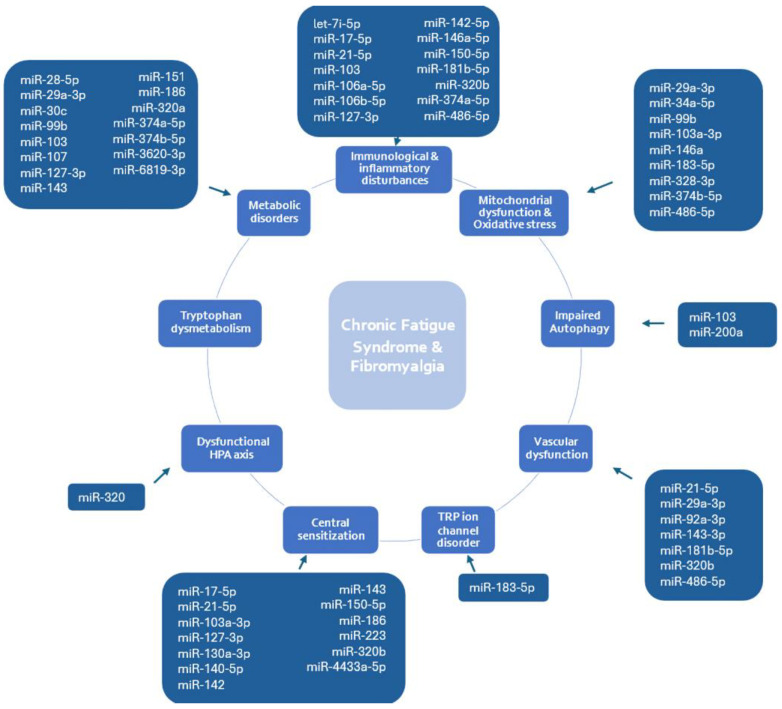
Overview of miRNAs implicated in dysregulated processes manifesting myalgic encephalitis/chronic fatigue syndrome (ME/CFS) and/or fibromyalgia (FM) that were reported at least two times in different research studies.

A compelling body of evidence has supported the implication of altered expression of miRNAs in ME/CFS and/or FM syndromes [83,84,85,86]. The identified miRNAs from relevant studies in the literature (Table S1 in Supplementary Materials) were further linked to the suggested dysregulated processes in these chronic diseases, accordingly. Figure 2 illustrates the most frequently cited miRNAs identified in at least two independent studies. Additionally, for each pathophysiological process, we highlight the miRNAs that were reported.

Considering the miRNAs that have been reported to be involved in immunological and inflammatory disturbances in FM and/or ME/CFS (*n* = 26), miR-17-5p [87,88,89], miR-21-5p [87,89,90], miR-127-3p [83,86,91,92,93], miR-146a-5p [87,88,89,94], miR-150-5p [83,86,88,93], and miR-320b [95,96,97,98] were found to have the greatest number of mentions (>3) in different publications. Concerning the most frequently mentioned miRNAs (>3 publications) regulating mitochondrial dysfunction and oxidative stress (*n* = 21) in these chronic conditions, miR-29a-3p [83,93], miR-34a-5p [90,99], miR-99b [87,100], miR-103a-3p [89,95], miR-146a [87,88,89,94], miR-183-5p [88,91], miR-328-3p [91,101], miR-374b-5p [83,93,95], and miR-486 [83,93] were indicated. 

Among the autophagy-related miRNAs (*n* = 8), miR-103 [87,89,95] was most frequently reported as being dysregulated. Regarding the miRNAs implicated in vascular dysfunction (*n* = 26) in these conditions, miR-21-5p [85,87,89,90], miR-143-3p [86,91,92], and miR-320b [95,96,97,98] were the most frequently (>2) mentioned. 

In addition, with mentions in more than three publications, the most studied miRNAs involved in the central sensitization process (*n* = 29) in these diseases include miR-17-5p [87,88,89], miR-21-5p [85,87,89,90], miR-127-3p [83,86,91,92,93], miR-140-5p [83,86,88,93], miR-142 [86,92,95], miR-143 [86,91,92], miR-150-5p [83,86,93], and miR-320b [95,96,97,98]. As for the dysregulated miRNAs in metabolic disorders (*n* = 37) in ME/CFS and/or FM, miR-127-3p [83,86,92,93], miR-374b-5p [33,83,93,95], miR-107 [95,102], miR-143 [86,91,92], miR-320a [95,96,97,98], and miR-374b-5p [83,93,95] were identified in at least studies. Only miR-183-5p [91] was found to be involved in TRP ion channel disorder and miR-320(a) [95,96,97,98] in the dysfunctional HPA axis in these pain conditions, while no miRNAs were identified as being involved in tryptophan dysmetabolism in ME/CFS and/or FM. 

**Figure 3:**
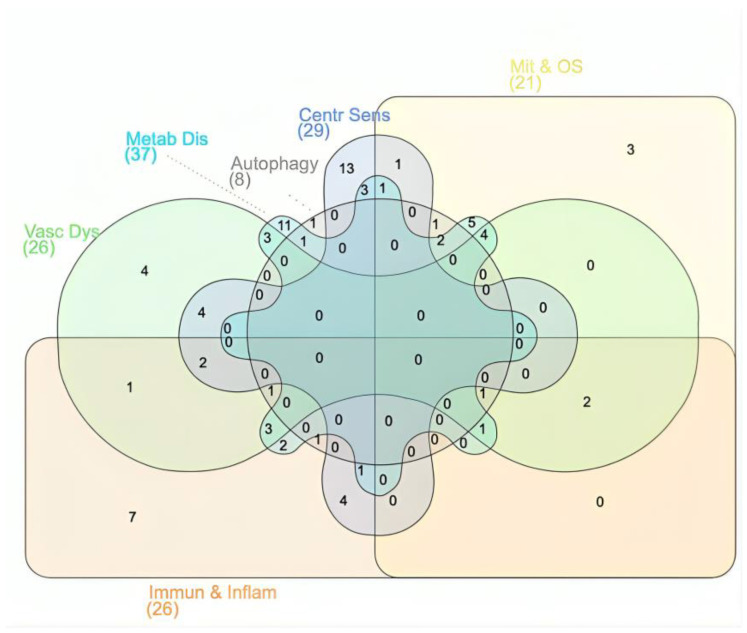
Overlapped miRNAs among the pathophysiological processes possibly manifesting fibromyalgia (FM) and/or myalgic encephalomyelitis/chronic fatigue syndrome (ME/CFS). Abbreviations: Metab Dis: metabolic disorder (*n* = 37 miRNAs), Centr Sens: central sensitization (*n* = 29 miRNAs), Immun & Inflam: immunological and inflammatory disturbances (*n* = 26 miRNAs), Vasc Dys: vascular dysfunction (*n* = 26 miRNAs), Mit & OS: mitochondrial dysfunction and oxidative stress (*n* = 21 miRNAs), Autophagy (*n* = 8 miRNAs). The InteractiVenn tool was used to generate the diagram [123].

The overlap of miRNAs among the pathophysiological processes in ME/CFS and/or FM (Figure 3) revealed that miR-152 was found to regulate most of the pathological processes manifesting these chronic diseases, including immunological and inflammatory disturbances, mitochondrial dysfunction, oxidative stress, impaired autophagy, and vascular dysfunction. In more detail, the significantly decreased expression of miR-152 was reported in the NK cells of ME/CFS patients compared to non-fatigued controls [89]. In that study, it was suggested that miR-152 may be involved in the reduced cytotoxic activity often reported in patients with ME/CFS, via targeting inhibitory human leukocyte antigen G (HLA-G). In another study, it was demonstrated that miR-152(-3p) regulated chronic-pain-induced depression-like behaviors by targeting DNA methyltransferase 1 (DNMT1) [103].

In addition, miR-23a was shown to be involved in immunological and inflammatory disturbances, mitochondrial dysfunction/oxidative stress, metabolic disorders, and vascular dysfunction. The downregulation of miR-23a has been reported in FM patients [98,104] compared to controls. This miRNA is a member of the miR-23a-27a-24-2 cluster, which is implicated in many diseases, including muscle atrophy via targeting muscle-specific F-box protein (MAFx)/atrogin-1 [105]. Also, miR-23b (from the same cluster) has been shown to regulate the expression of μ-opioid receptors [106] and to be underexpressed in various autoimmune diseases via suppressing interleukin 17 (IL-17), tumor necrosis factor α (TNF- α), or IL-1β-induced NF-kB activation [107]. 

Moreover, miR-320 was implicated in the regulation of the HPA axis, mitochondrial function, oxidative stress, inflammation, and metabolic disorders. In patients with FM, miR-320a has been reported to be upregulated [95,108], while miR-320b was downregulated [98] compared to healthy controls. It has been shown that miR-320 targets genes involved in pain disorders, particularly by binding to FK506 binding protein 5 (FKBP5) [109], which plays an important role in the HPA axis and subsequently, in pain processing and the stress response. Additionally, miR-320a has been shown to regulate neuroinflammation by inhibiting its targets, transforming growth factor-beta receptor 2 (TGFBR2) and SMAD Family Member 2 (SMAD2), resulting in increased inflammatory cytokines, mitochondrial dysfunction, apoptosis, and oxidative stress [110,111]. Finally, miR-320(a) has been shown to target IGF-1 receptor and/or IGF-1 [111,112]. 

MiR-103(a) was involved in the immune inflammatory response, mitochondrial dysfunction and oxidative stress, impaired autophagy, central sensitization, and metabolic disorders. In FM patients, miR-103a-3p was underexpressed compared to healthy controls and was associated with pain and sleep quantity [104]. In patients with ME/CFS, the expression of miR-103 was lower in NK cells in comparison to the non-fatigued controls [89]. MiR-103 has been suggested as a therapeutic target, as its downregulation led to hypersensitivity to pain via the regulation of L-type calcium channel (in an animal model) [113].

Five miRNAs, namely, miR-29c-5p, miR-99b, miR-128-3p, miR-374b-5p, and miR-766, were found to be commonly implicated in metabolic disorders and mitochondrial dysfunction/oxidative stress. MiR-29c is a member of the miR-29 family, which has a key role in metabolism and metabolic disease [114], among numerous other human diseases [115]. MiR-29c targets the muscle atrophy genes muscle RING-finger protein-1 (MuRF1), Atrogin-1, and histone deacetylase 4 (HDAC4) and has been associated with skeletal muscle size and function [116]. Overexpressed miR-99b in blood and NK cells in patients with ME/CFS was previously reported [100], suggesting an mTOR signaling pathway and, subsequently, a reduction in NK cell cytotoxic and cytokine effector function [117,118]. The upregulation of miR-128-3p has been reported in patients with FM [91]. MiR-128-3p suppresses bone formation via sirtuin 6 (SIRT6) [119] and targets the cholinergic system in FM [91]. It has also been implicated in the neuronal oxidative stress response [120]. MiR-374b-5p was found to be underexpressed in both FM [104] and ME/CFS patients [83] and is implicated in induced pain perception. MiR-374b-5p targets vascular endothelial growth factor A (VEGFA), involved in vasculogenesis and angiogenesis, which regulate the capillary supply in skeletal muscle and thereby may be linked to the post-exertional malaise and fatigue seen in patients with ME/CFS and FM [121]. MiR-766-3p was underexpressed in blood samples collected from patients with FM [91]. In addition, miR-766 regulated multiple pathways including apoptosis, extracellular matrix, and inflammatory reactions in human intervertebral disc degeneration [122].

### 3.3. Sex- and Age-specific Patterns 

There is around a three and six times higher prevalence of ME/CFS and FM in females, respectively [124]. Female sex is the most reproducible risk factor for both ME/CFS and FM [125,126], and the rates seem to increase with age [125,127,128]. The perception of pain has been shown to be different between biological men and women, with the latter exhibiting remarkably lower pain thresholds, which could be possibly and partially attributed to their different hormonal profiles [129,130]. Presumably, the differences in gender and/or sex that affect hormones and immune responses can also influence susceptibility in chronic pain conditions [131]. Moreover, hormonal fluctuations during the menstrual cycle and reproductive phase have also been reported to be involved in chronic pain disorders [132]. Among gonadal steroid hormones, estrogens have been shown to regulate nociceptive pathways and, subsequently, pain via targeting intracellular receptors such as GPCRs of the CNS and peripheral nervous system, and modulating serotonergic, noradrenergic, dopaminergic, and endogenous kappa (k) or μ-opioid pathways [129,133,134,135,136]. Estrogens modulate the synthesis and metabolism of serotonin, the dysregulation of which has been associated with pain disorders [137]. In contrast, androgens, which seem to inhibit the expression of estrogen receptors, act as a nociceptive protective agent against stresses [138]. Low levels of adrenal androgens, particularly serum concentrations of neurosteroids and dehydroepiandrosterone sulphate (DHEAS), have been observed in both ME/CFS and FM [139]. Women in a high-estrogen state showed decreased pain sensitivity, increased brain mu-opioid receptor binding, and higher levels of activated endogenous opioid neurotransmission during pain, whereas women in a low-estradiol state exhibited significantly decreased endogenous opioid neurotransmission associated with hyperalgesia [135].

Besides sex and/or gender, age is also a contributing factor for acquiring ME/CFS [140] and FM [141]. Two peaks in the incidence of ME/CFS, between 10 and 19 years and between 30 and 39 years, have been described [139]. In another study, older age was associated with the risk of severe fatigue syndrome, but not with the risk of developing ME/CFS [142]. Higher fatigue scores were observed in older patients with ME/CFS compared to younger ones [143]. The impact of age on the development of FM has been possibly attributed to the observed decrease in gray matter, which, in turn, has been associated with pain sensitivity [144]. It has been demonstrated that patients with FM experienced an age-related reduction in gray matter at a rate 3.3 times faster than that of healthy individuals, with each year of the FM equating to 9.5 times the normal aging loss in terms of gray matter [145]. In addition, increasing age was associated with an increase in symptom duration, but a decrease in FM symptoms [141]. In particular, decreased pain and depressive symptoms have been reported in older patients with chronic pain conditions compared to younger patients [146]. 

## 4. Conclusions

Chronic-fatigue-related syndromes, such as ME/CFS and FM, share an overlapping clinical picture, described by common symptoms. Although several risk factors have been described, the pathophysiology underlying these syndromes is not well defined, impeding their accurate diagnosis and effective treatment. Many dysregulated processes have been implicated in both diseases. A better understanding of the mechanisms involved in these diseases may lead to the identification of biomarkers, such as some of the miRNAs discussed in this review. Within this context, the identification of miRNAs implicated in these chronic diseases can be valuable for the timely diagnosis of FM and ME/CFS. Filling this gap can certainly benefit not only patients’ quality of life, but also future drug discovery for these chronic conditions.

## 5. Future Directions

In this review, several miRNAs, commonly dysregulated in pathophysiological processes, were identified using existing human data from the literature. These miRNAs appear to play potential roles in various biological processes such as immune response, inflammatory disturbances, mitochondrial dysfunction, oxidative stress, autophagy, vascular dysfunction, central sensitization, and metabolic disorders. This miRNA-based approach provides a clear direction for future research and potential clinical applications. These findings may also provide therapeutic targets. In most of our reviewed studies, a higher percentage of participants were women, as is true of most studies of ME/CFS and FM. Another significant strength of our study is its focus on miRNAs as molecular biomarkers, which are known to be extremely stable due to their short length, particularly in biofluids collected by minimally invasive methods. These miRNAs may serve as sensitive indicators of pathological processes, contributing to early diagnosis and targeted therapeutic interventions.

Nevertheless, this study has also its limitations. Our research relied on published studies, which may introduce publication bias and may overestimate the importance of certain miRNAs. In addition, these studies are often cross-sectional, making it difficult to draw conclusions about the causality of the relationship between miRNA dysregulations and the development of or progression of these conditions. In addition, confounding and lifestyle factors may also influence the interpretation of miRNA dysregulations. Lastly, while the study identifies potential miRNA biomarkers, further clinical validation is necessary to confirm their efficacy and reliability in diagnosing ME/CFS and FM.

## Supplementary Materials

The following supporting information can be downloaded at: https://www.mdpi.com/article/10.3390/ijms25179551/s1 [147,148,149,150,151,152,153,154,155,156,157,158,159,160,161,162,163,164,165,166,167,168,169,170,171,172,173,174,175,176,177,178,179,180,181,182].
